# Novel Coronavirus Disease 2019 Infection in Children: The Dark Side of a Worldwide Outbreak

**DOI:** 10.3389/fped.2020.00215

**Published:** 2020-04-30

**Authors:** Danilo Buonsenso, Giuseppe Zampino, Piero Valentini

**Affiliations:** ^1^Department of Woman and Child Health and Public Health, Fondazione Policlinico Universitario A. Gemelli IRCCS, Rome, Italy; ^2^Istituto di Pediatria, Università Cattolica del Sacro Cuore, Rome, Italy

**Keywords:** coronavirus, COVID-19, children, public health, SARS-CoV-2

## Abstract

In this pediatric perspectives article, we discuss current limits in the understanding of novel coronavirus infection. In our opinion, the burden of novel coronavirus infections is underestimated because not actively looked for. We discuss the basis of our observations and what this can generate, suggesting a different approach for the search of the virus in children.

## Introduction

On December 2019, a novel coronavirus, named Severe Acute Respiratory Syndrome Coronavirus 2 (SARS-CoV-2) emerged in Wuhan City, Hubei Province, China, causing a cluster of pneumonia cases (1). Since then, the infection has spread worldwide causing more than a million cases and thousands of deaths (https://gisanddata.maps.arcgis.com/apps/opsdashboard/index.html#/bda7594740fd40299423467b48e9ecf6).

To date, we know that individuals of all ages are susceptible to SARS-CoV-2, and that the elderly and those with underlying comorbidities are more likely to develop complications. However, in spite of the growing amount of data available on the SARS-CoV-2, a clear understanding of how SARS-CoV-2 affects children is still missing.

## Main Epidemiological Data on SARS-CoV-2 Infection in Children

The Novel Coronavirus Pneumonia Emergency Response Epidemiology Team reported the occurrence of a series of 72,314 cases. The authors describe incident infection in 416 children aged 0–10 (0.57% of all cases) with no reported fatality, and 549 adolescents (0.76%) aged 10 to 19 with one death reported (0.1% of all patients aged 19 years or less) (1). However, no further details were provided on this pediatric population. We do not know about their comorbidities, need for admission or invasive procedures. Other case reports (mostly in Chinese language) are providing similarly reassuring information on the outcomes of Coronavirus Disease 2019 (COVID-19) in children (2, 3).

However, The Lancet reported a family cluster of SARS-CoV-2 infections in which an asymptomatic child developed ground-glass opacities as seen on a CT scan, performed because of parents' pressure (4). This finding, although generally unnoticed by the experts, highlights an important point: children can be asymptomatic, but they can nonetheless develop lung abnormalities. This data can suggest that several other asymptomatically infected children might have developed lung disease. In that case, concerns regarding their viral load and spreading into the community should arise.

Recently published studies are providing a better understanding of the SARS-CoV-2 infection and natural history. Zou and colleagues (5) analyzed the nasal and pharyngeal viral load in 17 symptomatic adult patients. Higher viral loads were detected soon after the onset of symptoms, with higher viral loads detected in the nose than the throat. Importantly, a comparable viral load was detected in asymptomatic patients tested, suggesting the possibility of transmission from asymptomatic or paucisymptomatic patients. Similarly, Rothe et al. (6) reported the first probable case of SARS-CoV-2 transmission in Europe occurring from an asymptomatic adult during the incubation period. Recently, first data regarding viral load in children have been published. Cai et al. (7) described the SARS-CoV-2 infection in 10 children and confirmed a mild presentation and, importantly, documented prolonged virus shedding from both the respiratory tract and the feces during the convalescent stage. Also Xu et al. documented the fecal spreading of SARS-CoV-2 in children (8). Importantly, they found that fecal shedding lasted more than the nasopharyngeal one also in asymptomatic children, suggesting that thay can play a role in the spread of the infection, as they do with other respiratory viruses.

## Discussion

These observations have several implications, particularly in regard to the daily pediatric practice. To date, SARS-CoV-2 infection in children is not bearing attention and interest because it seems not clinically relevant. However, as pediatricians we are aware that children are extremely prone to contract and spread viral infections. Since the SARS-CoV-2 test is performed only to symptomatic patients or known contacts, how can we ensure that the small number of documented pediatric infections is not a huge underestimation? Moreover, the isolation of common respiratory viruses from children's nasal swabs is very frequent and, in the current circumstances, we could have mistakenly confirmed an exclusive etiological diagnosis without suspecting SARS-CoV-2 as an additional threat. We know little about potential coinfections of SARS-CoV-2 and other more common respiratory viruses.

Another consideration to bear in mind is that children are those that we all hug and kiss the most. Children spend much of their free time with the grandparents, which appear to be the subjects affected the most by the SARS-CoV-2 infection. Are we sure that children, although not significantly clinically involved, are not playing a role in the SARS-CoV-2 diffusion? To date, epidemiological data and studies in these groups are lacking, and worldwide guidelines suggest to test only symptomatic or suspected cases, without mentioning the necessity to test children that, although being asymptomatic, have possibly a hidden burden of infection.

This would not be the first time children are neglected during an outbreak of infectious diseases. A similar situation has been happening, for the past decades, with pediatric tuberculosis, defined as “neglected disease” and “human right violation” (9) by major experts: pediatric tuberculosis receives little attention and interest because it is usually paucisymptomatic and paucibacillary in children (therefore, has a milder impact on human population and economies). However, the epidemiology of this disease in the pediatric population plays a key role for a good understanding of the real tuberculosis burden and diffusion within the population. Is a similar phenomenon happening with pediatric SARS-CoV-2 infection?

It is possible that testing children would raise the number of confirmed cases dramatically, with catastrophic economic consequences. Would world health authorities and national governments commit to a better understanding of the epidemiology of the pediatric SARS-CoV-2 infection, at the dangerous cost of a rise of detected cases, fear in the general population, national and international restrictions, and heavy economic consequences?

In the developed countries where SARS-CoV-2 is currently spreading, the survival of children with severe morbidities, immune disorders, cancer, or transplant is much higher than other countries (including China) due to high-standards of medical care. We could suspect that the infection of compromised children carried out by peers, potential asymptomatic carriers, could lead to serious consequences. Although fatal cases have not yet been reported in the pediatric population, we cannot exclude that the vulnerable children have not been infected yet. To prevent the contagion of children at increased risk, a better understanding of the dynamics of SARS-CoV-2 among children is necessary.

China and Italy first, and subsequently many other countries in the world, have currently closed all schools, from primary nurseries to universities. This choice was based on the concern for aggregation and not on scientific evidence of a child-to-child transmission. Further information on transmission dynamics are needed, as well as a good understanding of the real burden of SARS-CoV-2 infection in children, in order to improve our screening practices. Children in high-burden areas should be tested and results correlated with their recent medical history as well as with infectious status of close contacts, in order to understand how and how long they shed the virus. Moreover, the better knowledge of the pediatric SARS-CoV-2 infection can clarify the underlying mechanisms that lead children to develop paucysymptomatic disease; this, in turn, can help researchers in findings novel therapeutic strategies against viral infections in general, and COVID-19 in particular. Recenlty, Spanish researchers evaluated a large series of children evaluated in 30 tertiary hospitals during the first 2 weeks of the epidemic in Spain (10). Although they found that only 41 of the 4,695 confirmed cases (0.8%) were children younger than 18 years of age, they only tested those evaluated in hospitals. This is an important limitation since most children are expected to be asymptomatic or have mild symptoms (11), and to know the real burden of pediatric COVID-19 and how this influences the SARS-CoV-2 diffusion, we need to test children in the community, in both high and low SARS-CoV-2 burden settings and compare results. Importantly, children should be tested with both nasopharyngeal and rectal swab, since authors state that pharyngeal and nasal swab sensitivity is as low as 32–63% (12). More importantly, serological studies are necessary to understand the real burden of pediatric COVID-19. We need to know that and stop neglecting and not considering children, this will really help in understanding epidemics in general (not only the current one) and knowledge gained will help to better manage the next one, which will come soon or later.

In conclusion, according to the current estimates, about 1% of all confirmed SARS-CoV-2 cases involve children. This could be an underestimation, as SARS-CoV-2 is not actively searched in children. There are still many open questions about the epidemiology of SARS-CoV-2 infection in children.

## Data Availability Statement

The original contributions presented in the study are included in the article/supplementary materials, further inquiries can be directed to the corresponding author.

## Author Contributions

DB conceptualized and designed the study, drafted the initial manuscript, and reviewed and revised the manuscript. DB and PV carried out literature review. PV and GZ critically reviewed the manuscript for important intellectual content. All authors approved the final manuscript as submitted and agreed to be accountable for all aspects of the work.

## Conflict of Interest

The authors declare that the research was conducted in the absence of any commercial or financial relationships that could be construed as a potential conflict of interest.

## Abbreviations

COVID-19: COronaVIrus Disease 2019
SARS-CoV-2: Severe Acute Respiratory Syndrome Coronavirus 2.
